# A Case of Vicarious Menstruation

**Published:** 1878-06

**Authors:** 


A Case of Vicarious Menstruation.—The patient is a laundress; she was born in New Hampshire, is twenty-four years old and single. Her catamenia began at nine, were characterized by moderate molimina, considerable flow, recurred regularly every four weeks, and continued three days.

During the summers of 1874 and 1875, it was her custom to spend one hour daily in a public bath, regardless of her catamenia. One day after the usual bath her menstrual flow suddenly and prematurely ceased; at the same time she was attacked with vertigo, distressing pains in the stomach and bowels, and palpitation of the heart. These symptoms, however, soon passed away. In four weeks she began to have lassitude, an inordinate craving for food, lancinating pains throughout the abdomen, with more or less tormina, followed by a sense of fullness of the stomach, pains extending from the temples to the vertex, fœtid breath, bad taste, and nausea. The latter symptom was quickly followed by her vomiting about a pint of dark grumous blood and ingesta. After the vomiting there was considerable prostration and febrile movement, which, with the earlier symptoms, gradually abated, and in three days left her "as well as ever." For two years these attacks of hæmatemesis continued to recur about the last of every month, preceded and followed by substantially the same train of symptoms that characterized the first. There was generally a slight amount of dysuria, which, however, disappeared with the other symptoms. The alvine dejections were normal, so far as could be ascertained.

There were no hereditary tendencies to disease, neither had the patient ever been seriously ill. When first seen by the reporter, she complained of fleeting pains in the chest, poor appetite, and malaise, but her general appearance was good.

An examination of the heart revealed nothing remarkable; neither did percussion nor auscultation of the chest elicit any but normal sounds.

There was no tumor or undue tenderness detected in the epigastrium.

The patient was given tonic treatment, and soon after left the institution, but returned a few weeks since, and now reports that seven months ago, while taking wine and iron, her catamenia returned, and have since recurred regularly, unaccompanied by any abnormal symptom. A physical examination resulted as before. She is in excellent health, and weighs thirty pounds more than she did one year ago.—*Boston Medical and Surgical Journal.*

